# Enhancing physiological metrics, yield, zinc bioavailability, and economic viability of *Basmati* rice through nano zinc fertilization and summer green manuring in semi–arid South Asian ecosystem

**DOI:** 10.3389/fpls.2023.1283588

**Published:** 2023-10-31

**Authors:** Kirttiranjan Baral, Yashbir Singh Shivay, Radha Prasanna, Dinesh Kumar, Cherukumalli Srinivasarao, Sunil Mandi, Somanath Nayak, Kadapa Sreenivasa Reddy

**Affiliations:** ^1^ Division of Agronomy, Indian Council of Agricultural Research (ICAR)-Indian Agricultural Research Institute, New Delhi, India; ^2^ Division of Microbiology, Indian Council of Agricultural Research (ICAR)-Indian Agricultural Research Institute, New Delhi, India; ^3^ Indian Council of Agricultural Research (ICAR)-National Academy of Agricultural Research Management, Hyderabad, Telangana, India; ^4^ Division of Natural Resource Management, Indian Council of Agricultural Research (ICAR)-Indian Agricultural Research Institute, Dhemaji, Assam, India

**Keywords:** economics, green manuring, growth, nano Zn, yields, zinc biofortification

## Abstract

During the summer and rainy seasons (April-October) of 2020 and 2021, two consecutive field experiments were conducted at the research farm of the ICAR-Indian Agricultural Research Institute, New Delhi, India. In this study, we examined the effects of summer green manuring crops (GM) and a variety of zinc fertilizers (ZnF) on *Basmati* rice (*Oryza sativa* L.) growth, physiological development, yield response, zinc nutrition and economic returns. A combination of GM residues and nano zinc fertilization helped significantly enhancing *Basmati* rice’s growth and its physiological development. Following the incorporation of *Sesbania aculeata* (Sesbania), successive *Basmati* rice physiological parameters were significantly improved, as well as grain, straw, biological yields, harvest index and economic returns. The highest Zn content of 15.1 mg kg ^-1^ and the lowest of 11.8 mg kg ^-1^ in milled rice grain were recorded in *Sesbania* green manuring (G2) and control i.e., in the fallow (G1), respectively. Coating onto urea with 0.2% nano zinc oxide (NZnCU) was observed to be more effective than other zinc sources in terms of growth parameters, yield attributes, zinc nutrition, grain and straw yields for succeeding *Basmati* rice crop; however, the effects were comparable to those of bulk zinc oxide-coated urea (BZnCU) of 1%. The highest Zn content of 15.1 mg kg ^-1^ was recorded with the application of 1% BZnCU and the lowest of 11.96 mg kg ^-1^ with the soil application of 5 kg Zn ha ^-1^ through bulk ZnO in the milled rice grain. Application of 1% BZnCU led to a 26.25% increase in Zn content of milled rice grain compared to soil application of 5 kg Zn ha ^-1^ through bulk ZnO. As a result, the combination of inclusion of *Sesbania aculeata* (*Sesbania*) residue and 0.2% NZnCU was identified as the most effective treatment, for Basmati rice growth and physiological development. A combination of nano Zn fertilization in conjunction with the incorporation of green manure can be advocated for better growth, physiological performance, zinc dense grains, and higher profitability of Basmati rice for farmers and consumers.

## 1 Introduction

India holds a prominent position as the world’s second–largest producer of rice, responsible for a fifth of global rice cultivation (Kumar et al., 2021). It serves as a vital nutritional source, fulfilling 15% of protein requirements and 21% of caloric needs for its populace (Prasad et al., 2017). Particularly noteworthy is the *Basmati* rice, which is prized for its elongation upon cooking and its fluffy texture. In the financial year 2021–22, the country garnered a staggering US $3.54 billion through *Basmati* rice exports alone, making it the global leader in this market (APEDA, 2023). However, the average yield of rice in India lags at 3.42 tons per hectare (Directorate of Economics and Statistics, 2020). Given the burgeoning population, the nation faces the daunting challenge of ramping up rice production to an estimated 130 million tons by 2030 to maintain food security. This task is complicated by numerous obstacles, including inadequate irrigation systems, deteriorating soil quality, and imbalances in macro– and micronutrients (Bhatt et al., 2016). For example, high temperature and moisture content in paddy soils hasten the decay of organic matter (Mohammad et al., 2005), resulting in its depletion. The use of green manures has been touted as a way to mitigate soil erosion and augment both soil structure and fertility (Mandal et al., 2003). One specific micronutrient issue that prevails, especially in Indian and South Asian paddy fields, is zinc (Zn) deficiency, observed in approximately 51.2% (Shukla et al., 2021) of Indian soils. The scarcity of zinc is exacerbated due to its transformation into insoluble compounds in waterlogged conditions and high soil pH (Prasad et al., 2014). Zinc is indispensable for various plant metabolic functions, ranging from cell wall synthesis to carbohydrate metabolism (Broadley et al., 2007). Its deficiency manifests as leaf discoloration, reduced growth, and sterile seeds (Cakmak, 2008).

Traditional solutions involve zinc fertilization (Nayak et al., 2022), the efficacy of which varies depending on the type of fertilizer (Karak et al., 2005) and the rice genotype (Nayak et al., 2023). Recently, nano zinc applications have gained traction, offering benefits such as greater efficiency (Sadhukhan et al., 2021), quicker response, and enhanced grain enrichment (Baral et al., 2019; Baral et al., 2020; Baral et al., 2023). Nano–zinc oxide (ZnO) application has been shown to bolster both rice yield and grain zinc content (Pavithra et al., 2017). Similarly, zinc oxide–coated urea has displayed a favorable impact on yield and zinc content in both rice and wheat crops (Shivay et al., 2008). According to the findings of another study (Dimkpa et al., 2020), coating urea with nano–ZnO led to improved plant performance as well as increased zinc accumulation in wheat. Notably, existing research largely concentrates on the sole effects of ZnO nanoparticles (NPs) on plant health. Very few studies explore the synergistic benefits of coupling summer green manuring with nano–ZnO–coated urea. The integrated approach of utilizing both green manures and nano–fertilizers could offer a holistic solution, promising enhanced crop growth, physiological performance, yield, and economic returns.

In light of the aforementioned factors, a two–year field studies were conducted to scrutinize the effects of summer green manures and nanoscale zinc fertilizers on various parameters, such as growth, physiological indices, and economic viability under summer green manure–*Basmati* rice cropping sequence.

## 2 Materials and methods

### 2.1 Experimental site

Over the duration of two successive years, field studies were carried at the ICAR–Indian Agricultural Research Institute, New Delhi, India. The experiments were undertaken during the summer–rainy seasons of 2020 (first year of field study) and 2021 (second year of field study) in sandy clay–loam soil, specifically a typic *Ustochrept* soil type. The research farm of the ICAR–Indian Agricultural Research Institute is positioned at 28°38′ 24′′ N, 77°10′ 26′′ E and 228.6 meters above mean sea level. The region experiences an average annual rainfall of 650 mm, with more than 80% occurring during the south–west monsoon season from July to September. The mean annual evaporation of the site is approximately 850 mm. The experimental field at the institute farm has been traditionally managed using conventional methods, following a 4–year crop rotation scheme (**Table 1**). However, the fertilization management varied during two years of our field study, as outlined in **Table 2**. The initial soil analysis revealed that the experimental field had 171 kg ha^–1^ of alkaline permanganate oxidizable nitrogen (N) (Subbiah and Asija, 1956), 15.9 kg ha^–1^ of available phosphorus (P) (Olsen et al., 1954), 309 kg ha^–1^ of 1 N ammonium acetate exchangeable potassium (K) (Hanway and Heidel, 1952), and 0.65% organic carbon (C) (Prasad et al., 2006). The soil pH, determined using a 1:2.5 soil and water ratio, was found to be 7.68 (Prasad et al., 2006). Additionally, the diethylene triamine penta acetic acid (DTPA)–extractable zinc (Zn) content in the soil was measured to be 0.67 mg kg^–1^ (Lindsay and Norvell, 1978). Based on the critical level of DTPA–extractable Zn ranging from 0.38–0.90 mg kg^–1^ soil, for rice cultivation in the rice–wheat belt of North India, as reported by (Takkar et al., 1997), it was hypothesized that the application of Zn would affect the growth and yield of *Basmati* rice in the experimental field.

**Table 1:** Cropping history of the experimental field.

Year	*Kharif* season	*Rabi* season
2016–2017	Rice	Wheat
2017–2018	Rice	Wheat
2018–2019	Rice	Wheat
2019–2020	Rice	Wheat

**Table 2:** Details of experimental treatments applied.

(A) Main plot (Green manuring)	Treatment Description
Control (G1)	No green manuring i.e., the field was kept fallow
*Sesbania* (*Sesbania aculeata*) (G2)	It was incorporated into the soil at 45 days after sowing (DAS)
Cowpea (*Vigna unguiculata*) (G3)	It was incorporated into the soil at 45 DAS
(B) Sub–plot (Zn fertilizer sources)	
0 kg N + 5 kg Zn ha^–1^through bulk ZnO as soil application (control for N) [ZnF1]	5 kg Zn ha^–1^as bulk ZnO
N through prilled urea + no Zn (control for Zn) [ZnF2]	N at 120 kg N ha^–1^as prilled urea
N through prilled urea + 5 kg Zn ha^–1^ through bulk ZnO as soil application [ZnF3]	N at 120 kg N ha^–1^as prilled urea + 5 kg Zn ha^–1^as bulk ZnO
1% bulk ZnO–coated urea (1% BZnCU) [ZnF4]	N at 120 kg N ha^–1^as prilled urea + 2.08 kg Zn ha^–1^as bulk ZnO
0.1% nano ZnO–coated urea (0.1% NZnCU) [ZnF5]	N at 120 kg N ha^–1^as prilled urea + 0.208 kg Zn ha^–1^as nano ZnO
0.2% nano ZnO–coated urea (0.2% NZnCU) [ZnF6]	N at 120 kg N ha^–1^ as prilled urea + 0.416 kg Zn ha^–1^as nano ZnO

### 2.2 Treatment details

The experiment was conducted in a split–plot design with one summer fallow and two green manure crops as the main plot and six subplots comprising diverse Zn fertilizer sources with three replication. A detailed description of the treatments is given in **Table 2**.

### 2.3 Characterization of different Zn sources used in the experiment

The Zn sources employed in our experiment i.e., both bulk ZnO and nano ZnO (manufacturer: Sigma Aldrich) were characterized using the following techniques mentioned in **Table 3**.

**Table 3:** Details of characterization techniques deployed.

S. No.	Methods employed	Observations/Findings
1)	X–ray diffraction (XRD)	All the peaks were matching with the standard data of the hexagonal ZnO wurtzite structure (JCPDS card no. 36–1451). Hence, confirms the crystalline structure of both ZnO used
2)	Dynamic Light Scattering (DLS)	The 95% of the nano ZnO particles used in our experiment had a diameter of less than 74 nm, hence confirming the nano size of formulations i.e. less than 100 nm and it was more than 3430 nm (beyond 100 nm) as observed in bulk counterpart
3)	Scanning Electron microscopy (SEM) And Energy Dispersive X–ray spectroscopy (EDS)	Peaks for carbon, oxygen, and nitrogen were observed in the sole urea granules. The Zn peak was seen in all of the coated urea granules, in addition to the peaks seen in the uncoated granules. The granules that were not coated had no zinc
4)	High–resolution transmission electron microscopy (HRTEM)	The size of nanoparticles of ZnO was found well in the range of 1 to 100 nm

### 2.4 Crop establishment and management

The 45–day–old green manure crops were incorporated into the experimental field using a tractor–operated disc harrow, as per the specific treatment requirements. To ensure proper mixing, two rounds of harrowing were carried out. Before incorporating the green manure crops into the soil, a substantial irrigation of 10 cm was applied to the land. The field was then subjected to two rounds of disk ploughing, followed by three cycles of puddling using a puddler in the presence of standing water. At the final round of puddling, 26 kg P ha^–1^ in the form of single superphosphate and 33 kg K ha^–1^ in the form of muriate of potash were evenly distributed. For the application of nitrogen, prilled urea (manufacturer: KRIBHCO) and zinc–coated urea were utilized, which were divided into three equal portions. One–third of the nitrogen was applied as a basal dose, another third at the 50% tillering stage, and the remaining third at the panicle initiation stage. In both years of the study, during the first fortnight of July, the *Basmati* rice variety ‘Pusa Basmati 6’, aged 25 days was transplanted into the field at a spacing of 20 cm × 10 cm. Throughout the experiment, the rice crop was cultivated following the recommended package of practices. Detailed information regarding the schedule of crop establishment followed during both years of experimentation can be found in **Table 4**.

**Table 4:** Details of agronomic practices followed for *Basmati* rice.

S. No.	Observation	Date of operation	
		Year–2020	Year–2021
1)	Nursery bed preparation	09.06.2020	10.06.2021
2)	Sowing of nursery	11.06.2020	14.06.2021
3)	Final land preparation	05.07.2020	08.07.2021
4)	Preparation of layout	06.07.2020	09.07.2021
5)	Application of Basal Fertilizer	06.07.2020	09.07.2021
6)	Transplanting	07.07.2020 and 08.07.2020	10.07.2021 and 11.07.2021
7)	Gap filling	22.07.2020	24.07.2021
8)	Harvesting and drying	18–21.10.2020	21–24.10.2021
9)	Threshing, cleaning, drying, and weighing	24–27.10.2020	26–29.10.2021

### 2.5 Determination of physiological indices, yield and economics

Plant height for *Basmati* rice was measured at regular 30–day intervals until harvest using a standard metre scale and recorded in centimetres (cm) from the base of the plant at the ground surface to the tip of the tallest leaf. The amount of dry matter accumulated by *Basmati* rice on five different hills was monitored at 30–day intervals. After being air dried, these plants went into a hot air oven set to 60 ± 2°C to continue the drying process until a constant weight was achieved. The data was collected using a dry weight scale and expressed in g hill^–1^. The surface area of the leaves was determined by removing them from the plant, washing them in deionized water, and drying them on paper towels. Leaf area was measured using a leaf area metre (Model LICOR 3000, USA) and results were given in square centimetres per plant unit of foliage. The leaf area index (LAI) was determined at 30, 60, and 90 DAT using (Evans, 1972). To determine the crop growth rate (CGR) in a given area and over a given time period, the following formula (Watson, 1952) was used:


(1) $$\text{CGR }\left({\text{g m}}^{–2}{\text{day}}^{–1}\right)=\left(\text{W}2–\text{W}1\right)/\left(\text{T}2–\text{T}1\right)\times \left(\text{land area}\right)$$

Where, W1 and W2 are the dry weight (g) values at time T1 and T2, respectively. T1 and T2 are time in days after transplanting. The photosynthetic efficiency of leaves is measured by the net assimilation rate (NAR), which is the gain in assimilate per unit leaf area over time (Watson, 1952):


(2) $$\text{NAR}\left({\text{g m}}^{–2}{\text{day}}^{–1}\right)=\left(\text{W}2–\text{W}1\right)\;\left(\text{ln LA}2–\text{ln LA}1\right)/\;\left(\text{T}2–\text{T}1\right)\;\left(\text{LA}2–\text{LA}1\right)$$

Where, W1 and W2 are dry weights and LA1 and LA2 are leaf area values recorded at times T1 and T2, respectively. The RGR was also computed as per the procedure of (Watson, 1952).

The seeds were washed, dried in the sun, and weighed after threshing. The yields were transformed to tonnes per hectare. Before the plants were threshed, their weight was recorded after they had been dried in the sun. The yield of straw was calculated by subtracting the weight of the seeds from the total. The yields of both grain and straw were reported in tonnes per hectare. The market price of various inputs was used to estimate the cost of cultivation for various green manuring and Zn fertilization strategies. Net returns were determined by subtracting the cost of cultivation from the gross returns, which were determined by calculating the grain and straw yield and the prevailing market prices of grain and straw in respective seasons. By dividing net returns by the cost of cultivation, the benefit–cost ratio was computed.

### 2.6 Chemical analysis

Plant specimens harvested from the field were subjected to natural sun–drying, followed by oven–drying at a controlled temperature of 60 ± 2°C for a six–hour duration. Subsequently, the samples were pulverized. Analytical chemistry techniques were employed on 0.5–gram portions extracted from the rice grain and straw. The concentration of micronutrients in the dry matter was assessed utilizing di–acid digestion protocols, and quantification was performed through atomic absorption spectrophotometer (model: Elements AS AAS4141, ECIL, Hyderabad, India) (Prasad et al., 2006). Nutrient uptake metrics were then determined by multiplying these concentrations with the dry weight, with results expressed in grams per hectare (g ha^–1^).

### 2.7 Statistical analysis

A pooled analysis was performed on data collected over a span of two years, and the averages were reported. The statistical analyses were executed using R (version 4.2.2) in conjunction with R–Studio (version 2022.12.0 + 353), utilizing the “agricolae” package (de Mendiburu and de Mendiburu, 2019). To evaluate the statistical significance between different treatment groups, a 5% significance level was adopted. Further, Duncan’s Multiple Range Test (DMRT) was employed as a post–hoc analysis for categorizing treatment means (Gomez and Gomez, 1984).

## 3 Results

### 3.1 Growth and physiological indices of *Basmati* rice

**Figure 1:**
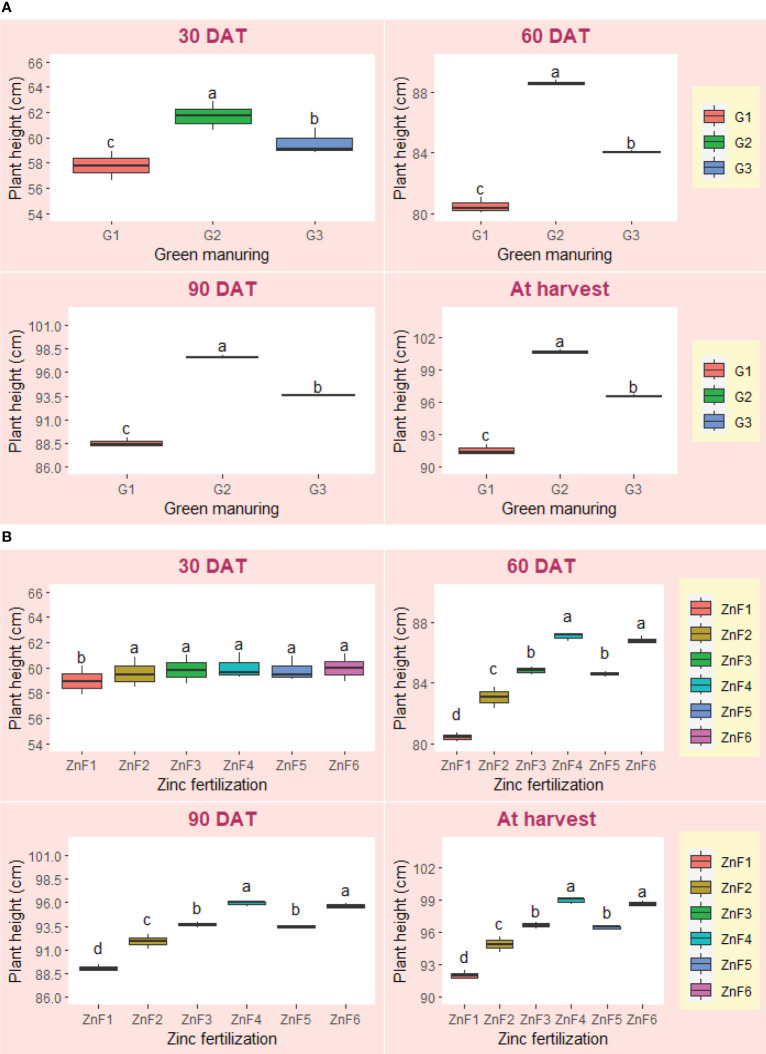
**(A)** Effect of summer green manuring on the plant height (cm) of *Basmati* rice (pooled data of 2 years). **(B) **Effect of Zn fertilizer sources on the plant height (cm) of *Basmati* rice (pooled data of 2 years). Boxplot of same letter(s) don’t differ significantly at 5% probability level by DMRT. DAT, Days after transplanting.

**Figure 2:**
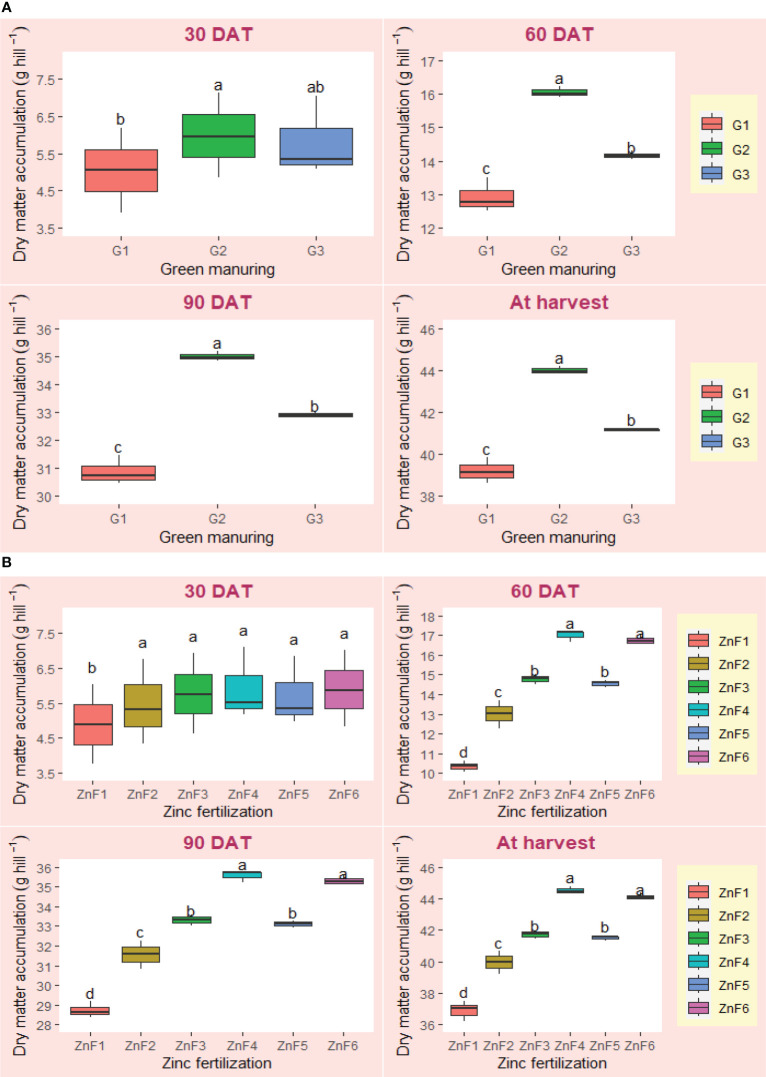
**(A)** Effect of summer green manuring on the dry matter accumulation (g hill^–1^) of *Basmati* rice (pooled data of 2 years). **(B)** Effect of Zn fertilizer sources on the dry matter accumulation (g hill^–1^) of *Basmati* rice (pooled data of 2 years). Boxplot of same letter(s) don’t differ significantly at 5% probability level by DMRT. DAT, Days after transplanting.

The impact of summer green manuring (GM) and zinc fertilization (ZnF) on various growth metrics of *Basmati* rice was found to be statistically significant, as evidenced in **Table 5** and **Figures 1**, **2**. Distinctive responses to GM were observed in relation to key parameters such as plant height, dry matter content, leaf area index, crop growth rate (CGR), and net assimilation rate (NAR) (**Table 5** and **Figures 1**, **2**). Among the GM crops, *Sesbania* (G2) yielded significantly taller plants compared to the cowpea GM treatment (G3) at the time of harvest, with the control group exhibiting the minimum plant height (**Figure 1**). *Sesbania* (G2) also surpassed other GM treatments in terms of dry matter accumulation at harvest (**Figure 2**) and leaf area index at 60 days after transplanting (DAT) (**Table 5**). Regarding CGR and NAR metrics, *Sesbania* (G2) demonstrated superior performance during the trial period (**Table 5**). While zinc fertilization strategies also yielded a significant effect, particularly on plant height and leaf area index, the 1% BZnCU (ZnF4) regime yielded higher values at both flowering and harvesting stages. Remarkably, the synergistic implementation of *Sesbania* GM (G2) and 0.2% NZnCU (ZnF6) yielded elevated CGR and NAR values between 30–60 DAT, and this was statistically comparable to results from 1% BZnCU (ZnF4) application (**Table 5**). On a comparative scale, *Sesbania* GM (G2) elevated the dry matter content by approximately 12.2% over the control group at the point of harvest. The growth and physiological traits of *Basmati* rice were markedly influenced by both the integration of green manure crop residues and different zinc fertilization approaches. Amongst various GM alternatives, *Sesbania* (G2) consistently outperformed others in key growth indicators like plant height, leaf area index, and dry matter content at almost all growth stages, except for leaf area index at 30 DAT. Additionally, cowpea GM (G3) also contributed positively to the growth metrics of the *Basmati rice* plants. Conversely, the least growth was observed when *Basmati* rice was grown following a summer fallow regime (G1). The hierarchy of efficacy among different zinc sources was as follows: 1% BZnCU (ZnF4) = 0.2% NZnCU (ZnF6) > 0.1% NZnCU (ZnF5) > nitrogen via prilled urea + 5 kg Zn ha^–1^as bulk ZnO (ZnF3) > nitrogen via prilled urea (ZnF2) > 5 kg Zn ha^–1^ as bulk ZnO (ZnF1).

**Table 5:** Effect of summer green manuring and Zn fertilizer sources on various physiological indices of *Basmati* rice (pooled data of 2 years).

Treatment	LAI			CGR (g m^–2^ day^–1^)		RGR (mg g^–1^ day^–1^)	NAR (g m^–2^ day^–1^)
Summer green manuring	30 DAT	60 DAT	90 DAT	0–30 DAT	30–60 DAT	30–60 DAT	30–60 DAT
G1	1.21^a^	5.41^a^	1.75^a^	4.59^b^	13.15^b^	31.20^a^	2.51^a^
G2	1.39^a^	5.89^a^	1.95^a^	5.44^a^	16.77^a^	32.98^a^	2.94^a^
G3	1.32^a^	5.72^a^	1.87^a^	5.30^ab^	13.91^b^	29.77^a^	2.53^a^
S/NS (*p*=0.05)	NS	NS	NS	S	S	NS	NS
Zinc fertiliser sources							
ZnF1	0.58^b^	4.95^b^	1.13^b^	4.45^b^	8.81^d^	24.68^c^	1.89^d^
ZnF2	1.17^a^	5.54^a^	1.72^a^	4.99^a^	12.54^c^	29.15^b^	2.37^cd^
ZnF3	1.46^a^	5.82^a^	2.01^a^	5.24^a^	15.01^b^	31.77^ab^	2.68^abc^
ZnF4	1.62^a^	5.99^a^	2.17^a^	5.39^a^	18.47^a^	35.55^a^	3.20^a^
ZnF5	1.42^a^	5.78^a^	1.97^a^	5.21^a^	14.71^b^	31.55^ab^	2.65^bc^
ZnF6	1.60^a^	5.96^a^	2.15^a^	5.37^a^	18.11^a^	35.19^a^	3.15^ab^
S/NS (*p*=0.05)	S	S	S	S	S	S	S

Means followed by the same letter(s) within a column do not differ significantly at 5% probability level at DMRT. DAT, Days after transplanting. S, Significant at p=0.05; NS, Non–significant at p=0.05.

### 3.2 Yields and harvest index of *Basmati* rice

**Figure 3:**
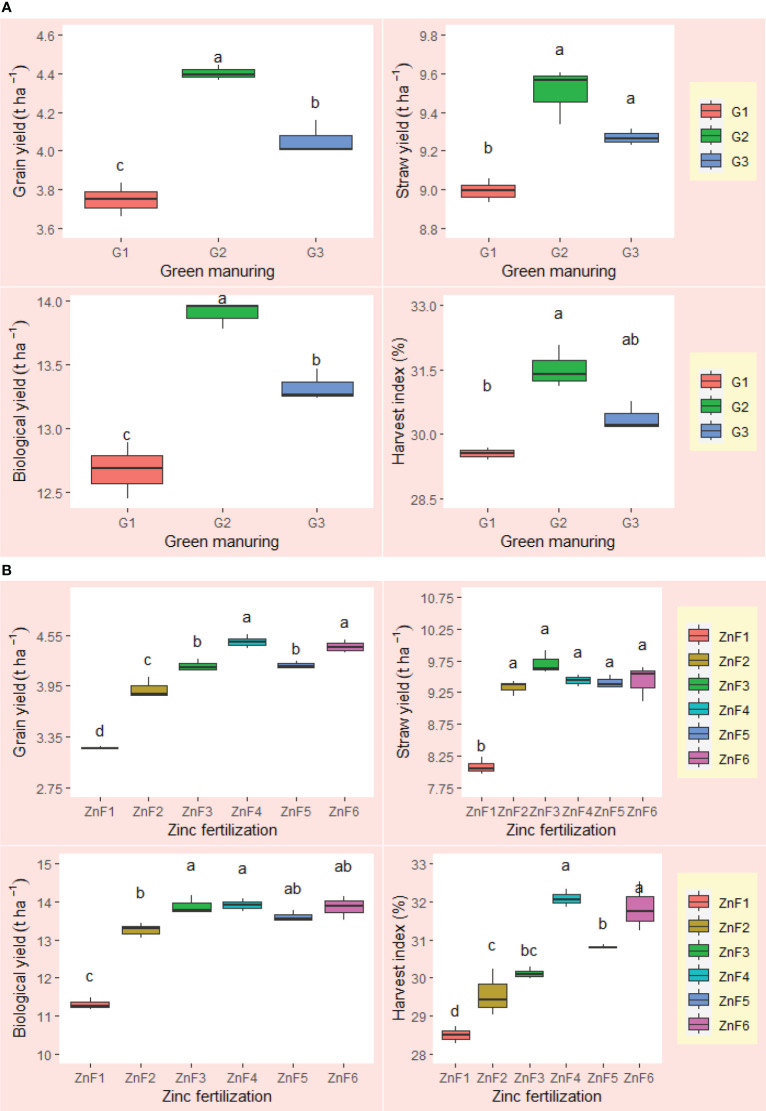
**(A)** Effect of summer green manuring on the yields and harvest index of *Basmati* rice (pooled data of 2 years). **(B)** Effect of Zn fertilizer sources on the yields and harvest index of *Basmati* rice (pooled data of 2 years). Boxplot of same letter(s) don’t differ significantly at 5% probability level by DMRT.

**Figure 4:**
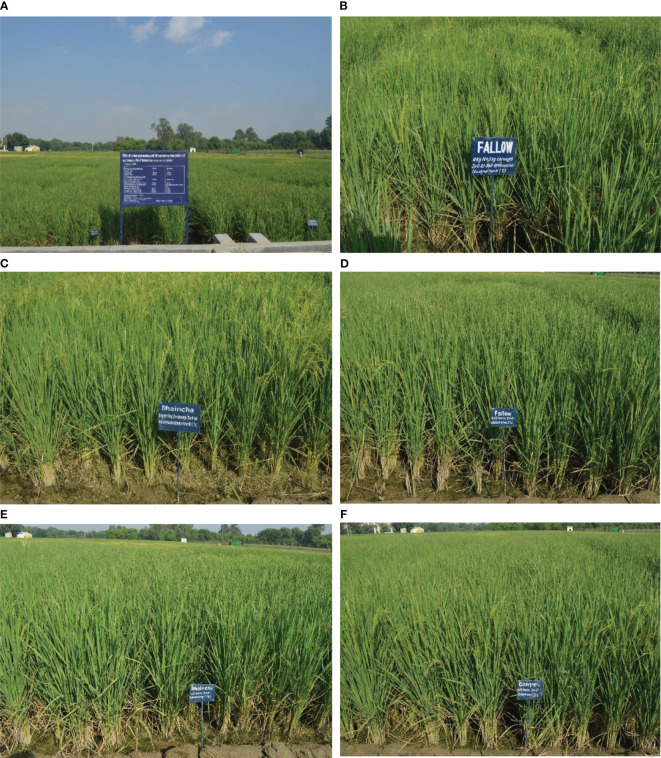
**(A)** Experimental field view. **(B)** Fallow+ 5 kg Zn ha^–1^ as bulk ZnO. **(C)** *Sesbania* + 5 kg Zn ha^–1^ as bulk ZnO. **(D)** Fallow + 0.2% NZCU. **(E)** *Sesbania* + 0.2% NZCU. **(F)** Cowpea + 0.2% NZCU.

**Figure 5:**
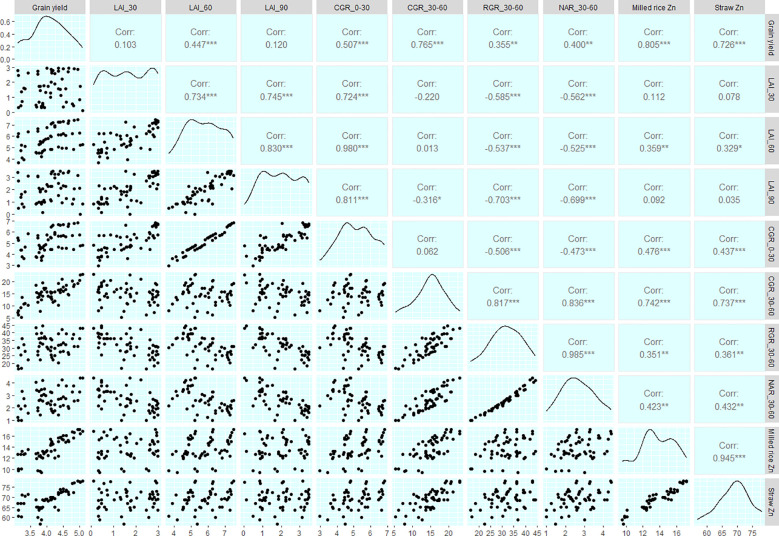
Correlation matrix depicting correlation co–efficient (upper half), density plot (diagonal) and scatter plot (lower half). LAI_30, Leaf area index at 30 DAT; LAI_60, Leaf area index at 60 DAT; LAI_90, Leaf area index at 90 DAT; CGR_0–30, Mean CGR 0–30 DAT; CGR_30–60, Mean CGR 30–60 DAT; RGR_30–60, Mean RGR 30–60 DAT; NAR_30–60, Mean NAR 30–60 DAT; DAT, Days after transplanting; *, Significant at *p*=0.05; **, Significant at *p*=0.01; ***, Significant at *p*=0.001.

**Figure 6:**
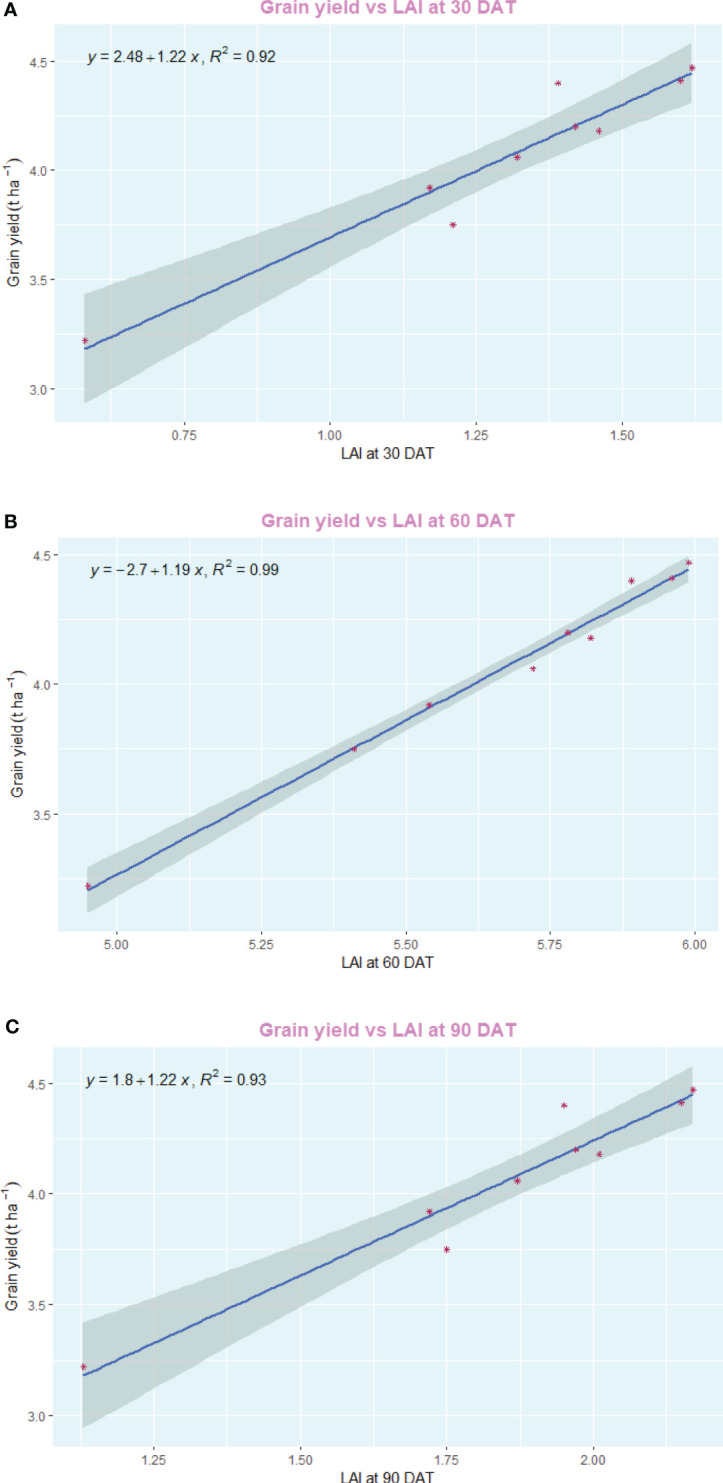
**(A)** Regression between grain yield and LAI at 30 DAT. **(B)** Regression between grain yield and LAI at 60 DAT. **(C)** Regression between grain yield and LAI at 90 DAT. DAT, Days after transplanting.

**Figure 7:**
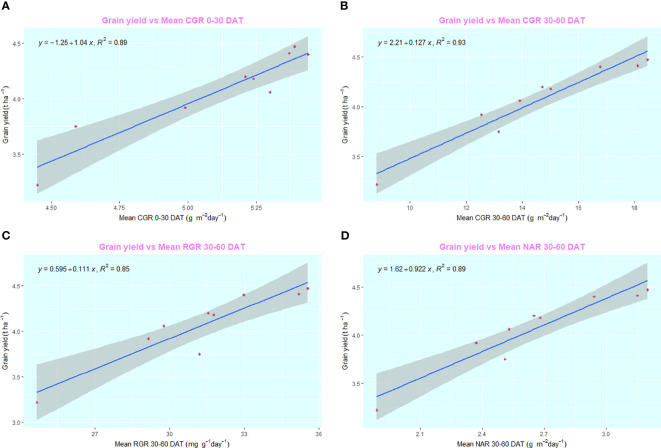
**(A)** Regression between grain yield and mean CGR 0–30 DAT. **(B)** Regression between grain yield and mean CGR 30–60 DAT. **(C)** Regression between grain yield and mean RGR 30–60 DAT. **(D)** Regression between grain yield and mean NAR 30–60 DAT. DAT, Days after transplanting.

**Figure 8:**
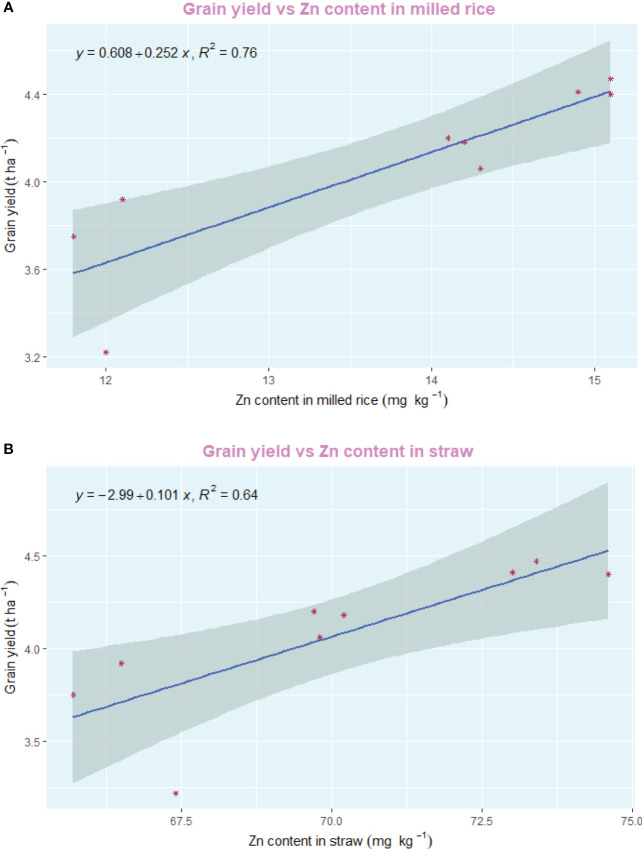
**(A)** Regression between grain yield and Zn content in milled rice. **(B)** Regression between grain yield and Zn content in straw.

The employment of nano zinc sources along with summer green manuring (GM) exhibited a pronounced influence on the yields of *Basmati* rice, as depicted in **Figures 3**, **4**. The incorporation of *Sesbania* (G2) as a green manure during the summer season led to significantly enhanced grain and straw yields, outperforming both cowpea (G3) and summer fallow (G1). Specifically, the 1% BZnCU (ZnF4) zinc fertilization regime delivered superior grain yield (4.47 t ha^–1^) and harvest index (32.1%), which were statistically on par with the outcomes from 0.2% NZnCU (ZnF6). The second most effective treatment was the 0.1% NZnCU application in terms of both grain and harvest index, as illustrated in **Figure 3**. In the context of performance metrics, the zinc sources manifested a hierarchical order of effectiveness: 1% BZnCU (ZnF4) = 0.2% NZnCU (ZnF6) > 0.1% NZnCU (ZnF5) > nitrogen supplied through prilled urea with 5 kg Zn ha^–1^ as bulk ZnO (ZnF3) > nitrogen supplied through prilled urea alone (ZnF2) > 5 kg Zn ha^–1^ as bulk ZnO (ZnF1), as shown in **Figure 3**. The application of 0.2% NZnCU (ZnF6) led to a grain yield and harvest index increase of 36.9% and 12.6%, respectively, over the control condition involving 5 kg Zn ha^–1^ as bulk ZnO. Correlation analyses between grain yield and various physiological indicators, as well as zinc content in the dry matter of both milled rice and straw, revealed intriguing patterns. A moderate but statistically significant linear correlation was observed between the leaf area index (LAI) at 60 days after transplanting (DAT) and grain yield (r=0.805, *p*=<0.001). Conversely, the correlations between grain yield and LAI at 30 DAT (r=0.078, p=< 0.05) and 90 DAT (r=0.035, p=<0.05) were not significant, as illustrated in **Figure 5**. Notably, there was a highly significant linear relationship between zinc content in the dry matter of straw (r=0.805, p=<0.001) and milled rice (r=0.726, p=<0.001) with grain yield (**Figure 5**). Subsequent regression analyses indicated that various factors accounted for significant variability in grain yield. Specifically, LAI at 60 DAT explained 99% of the variability (R²=0.99), followed by mean crop growth rate (CGR) 0–30 DAT (R²=0.89) and 30–60 DAT (R²=0.93), mean relative growth rate (RGR) 30–60 DAT (R²=0.85), mean net assimilation rate (NAR) 30–60 DAT (R²=0.89), and zinc content in the dry matter of milled rice (R²=0.76) and straw (R²=0.64), as elucidated in **Figures 6**–**8**.

### 3.3 Zinc nutrition

**Figure 9:**
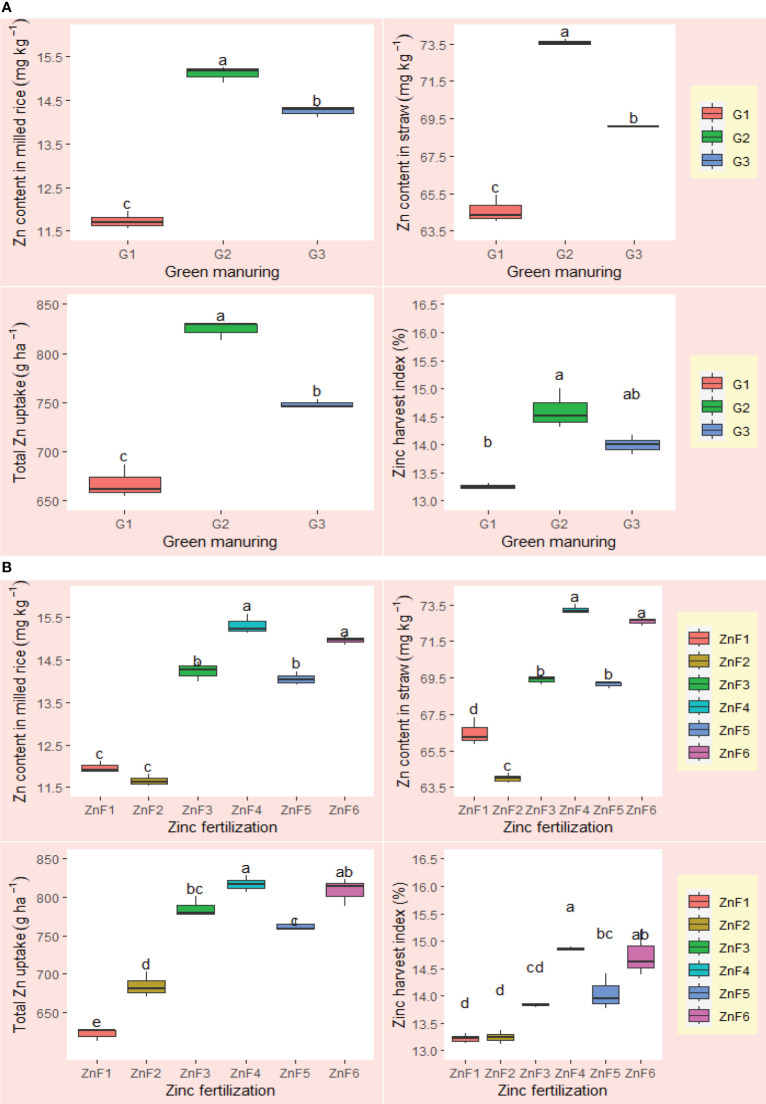
**(A)** Effect of summer green manuring on the zinc nutrition of *Basmati* rice (pooled data of 2 years). **(B)** Effect of Zn fertilizer sources on the zinc nutrition of *Basmati* rice (pooled data of 2 years). Boxplot of same letter(s) don’t differ significantly at 5% probability level by DMRT.

The investigation into the effects of green manures (GM) and zinc formulations (ZnF) on zinc nutrition of rice revealed that, marked variations were noted in zinc (Zn) concentrations within both milled rice and straw, overall zinc uptake, and the zinc harvest index (ZnHI). Across various GM applications, straw consistently outperformed milled rice in Zn concentrations (**Figure 9**), showcasing a range of 65.7–74.6 mg kg^–1^ in straw as opposed to a narrower 11.8–15.1 mg kg^–1^ spectrum in milled rice. In the realm of GM, *Sesbania* green manuring (G2) was identified as a superior performer, eclipsing fallow management (G1) in all measured Zn–related metrics across two years research period (**Figure 9**). The highest Zn content of 15.1 mg kg^–1^ and the lowest of 11.8 mg kg^–1^ in straw was recorded in *Sesbania* green manuring (G2) and control i.e., in the fallow (G1), respectively (**Figure 9**). When data were harmonized over various ZnF scenarios, G2 outperformed a noteworthy upswing in Zn metrics by 27.96%, 13.54%, 23.5%, and 10.18% in milled rice, straw, total Zn uptake, and ZnHI, respectively, relative to G1. In a parallel trend, cowpea green manuring (G3) also yielded Zn enrichment, albeit at a comparatively subdued scale. Strikingly, among the ZnF regime, 1% BZnCU (ZnF4) excelled, demonstrating comparable Zn enrichment capabilities with 0.2% NZnCU (ZnF6) in both grain and straw. The latter ZnF regime notably amplified Zn concentration in milled rice, straw, total Zn uptake, and ZnHI by increments of 24.16%, 8.30%, 30.0%, and 12.26%, respectively over sole application of bulk ZnO. The study further elucidated that an integrative approach, combining zinc with urea, either as a coating or through concurrent application, produced a synergistic uptick in Zn metrics. This conjoint strategy was notably more efficacious than singular urea applications or the standalone deployment of 5 kg Zn ha^–1^ via bulk ZnO. Such integrated methodologies bolstered Zn levels in milled rice, straw, total Zn absorption, and ZnHI by 20.41%, 7.62%, 15.91%, and 9.52%, respectively, over sole application of bulk ZnO.

### 3.4 Economics

In an economic analysis of *Basmati* rice cultivation, the financial metrics were notably influenced by the use of green manures and nano–zinc fertilization techniques (**Table 6**). Despite the premium seed cost associated with *Sesbania* green manuring (G2), this practice demonstrated better performance in delivering superior gross and net returns as well as an improved benefit–cost Ratio (net B:C) over the evaluation period. In particular, fields conditioned with *Sesbania* (G2) demonstrated superior yield performances compared to those with cowpea (G3) or fallow conditions (G1), thereby explaining its higher economic efficacy. Cowpea manuring (G3), although beneficial, lagged behind *Sesbania* in terms of all major economic indicators. Moreover, the financial robustness of *Basmati* rice cultivation was significantly swayed by different nano Zn fertilization regimes (**Table 6**). The application of 1% BZnCU (ZnF4) conspicuously helped in achieving noteworthy gross and net returns, as well as net B:C. Intriguingly, this performance was statistically indistinguishable from that of 0.2% NZnCU (ZnF6), with 0.1% NZnCU (ZnF5) regime is the second best. On deploying 0.2% NZnCU (ZnF6), a significant uptick was observed in both gross returns and net B:C by an average of 33.33% and 0.57, respectively than fallow.Consequently, a synergistic approach incorporating 0.2% NZnCU (ZnF6) fertilization coupled with *Sesbania* green manuring (G2) during the summer season presents a lucrative agronomic strategy for farmers grappling with zinc–deficient soils, offering a pathway to both sustainable productivity and optimized economic returns.

**Table 6:** Effect of summer green manuring and Zn fertilizer sources on economic returns of *Basmati* rice (pooled data of 2 years).

Treatment	Cost of cultivation (US $ ha^–1^)	Gross returns (US $ ha^–1^)	Net returns (US $ ha^–1^)	Net benefit: cost ratio
Summer green manuring				
G1	757.3	1684.5^c^	927.2^c^	1.22^b^
G2	814.8	1942.1^a^	1127.3^a^	1.38^a^
G3	826.3	1808.5^b^	982.2^b^	1.19^b^
S/NS (*p*=0.05)	–	S	S	S
Zinc fertiliser sources				
ZnF1	791.0	1463.0^d^	672.0^d^	0.85^d^
ZnF2	795.8	1760.7^c^	965.0^c^	1.21^c^
ZnF3	807.9	1871.0^b^	1063.0^b^	1.32^bc^
ZnF4	802.1	1965.8^a^	1163.7^a^	1.45^a^
ZnF5	798.7	1865.4^b^	1066.7^b^	1.33^b^
ZnF6	801.6	1944.6^ab^	1143^ab^	1.42^ab^
S/NS (*p*=0.05)	–	S	S	S

Means followed by the same letter(s) within a column do not differ significantly at 5% probability level at DMRT. S, Significant at p=0.05; NS, Non–significant at p=0.05.

## 4 Discussion

Peak leaf area index (LAI) values were observed at 60 days after transplantation (DAT), with value between 4.95 and 5.89. This leaf expansion is primarily due to the leaves reaching their full developmental and functional potential at this stage, eclipsing the leaf areas recorded at both 30 and 90 DAT. These observations are in harmony with previous research (Singh and Shivay, 2014). The differential treatment response can be linked to several factors: enhanced nutrient bioavailability, favorable modifications in soil physicochemical properties due to green manure infusion, and the elevated zinc bioavailability as a result of nano Zn fertilizer applications. These contributing factors likely accounted for the variations observed in plant growth metrics across different treatments. Additionally, the superior dry matter accrual observed in plants treated conjointly with urea and nano–zinc can be ascribed to the amplified LAI and crop growth rate (CGR) during the grain–filling phases. The vital role of zinc in chlorophyll biosynthesis has been corroborated by other studies (Sadak and Bakry, 2020). Evidence indicates that nano–Zn formulations enhance *Basmati* rice growth metrics due to their increased bioavailability compared to alternative zinc sources. Zinc, being critical for auxin synthesis, can influence plant height, as noted in research (Alloway, 2008). Furthermore, studies (Taran et al., 2017) have highlighted the stabilizing effect of ZnO nanoparticles on photosynthetic pigments, thereby boosting the plant’s photosynthetic capabilities. In the control plots, the stunted growth parameters could be attributed to soil submergence conditions, which precipitate the formation of insoluble zinc compounds, subsequently inhibiting plant zinc uptake and constraining growth. On the flip side, the superior zinc bioavailability in soils treated with 0.2% NZnCU (ZnF6) might result from limited soil interactions, thereby avoiding the common soil reactions seen with traditional zinc sources. The incorporation of *Sesbania* (G2) and cowpea (G3) green manures contributed dry biomass of approximately 5.2 and 3.43 t ha^–1^, and 4.98 and 3.24 t ha^–1^ in the first and second years of the study, respectively (**Supplementary Figure S1**). Particularly, *Sesbania*’s inclusion introduced readily decomposable organic matter, elevating the soil’s organic content and nutrient profile, which synergistically enhanced *Basmati* rice yield. The surge in grain and straw yields from the conjoint use of summer green manuring and nano–Zn fertilization can be elucidated through increased vegetative and physiological indices. For instance, the observed yield augmentation as a result of zinc fertilization (ZnF) is likely attributable to zinc–induced boosts in dry matter accumulation—evidenced by high LAI at 60 DAT—and other physiological parameters, as supported by a high R^2^ value in linear regression analyses. Moreover, this zinc–enhanced yield is also substantiated by the elevated zinc levels in both the straw and grain dry matter, further corroborated by high R^2^ values.

The current escalation in crop yield is closely linked to the zinc–induced augmentation of chlorophyll synthesis, corroborating findings from previous studies (Patel et al., 2022) that also attributed yield–enhancing attributes to improved photosynthetic efficiency. This study’s revelations, highlighting the efficacy of nano–zinc oxide in bolstering rice grain output, resonate with established literature on the subject (Kheyri et al., 2019; Adhikary et al., 2022). The amplification in both yield and harvest index in *Basmati* rice upon the utilization of nano–zinc–infused urea is likely driven by elevated zinc assimilation, subsequently fueling biomass expansion (Pooniya et al., 2012; Shivay and Prasad, 2012). Further, zinc’s role in facilitating the translocation of photosynthates amplifies these outcomes, aligning with earlier findings (Pavithra et al., 2017) that identified nano–zinc as a potent alternative for rice cultivation enhancement. Incorporating green manure crops prior to *Basmati* rice transplantation enriches the soil’s organic matter pool. This organic enrichment may have catalyzed the complexation of applied zinc with soil–bound organic compounds, thereby circumventing the formation of less bioavailable, insoluble zinc complexes. This leads to heightened zinc accessibility to the rice plants, culminating in superior growth and productivity metrics. Earlier research also substantiates the positive influence of zinc–fortified urea on plant physiology and yields (Shivay et al., 2008). The harmonious interplay between nitrogen and zinc availability likely triggered a synergistic crop response, eventually steering toward an elevated grain yield, a trend consistent with earlier research (Cahill et al., 2010). In the current analysis, zinc application was found to enrich the harvest index. Such improvements are arguably mediated by the zinc–induced surge in leaf area index, mean net assimilation rate (NAR) between 30 and 60 DAT, and overall dry matter accumulation. Comparatively, the study found ZnO NPs to be more efficacious than their bulk counterparts, with coated urea formulations also outpacing their uncoated equivalents. Typical coated fertilizers function as nutrient slow–release mechanisms, modulating the nutrient release rate from the encapsulating compound. On the other hand, non–coated urea tends to exhibit rapid nutrient release kinetics due to its higher solubility. Bulk ZnO displays quick dissolution upon aqueous contact (Shivay et al., 2008), whereas ZnO NPs, owing to their minute size and elevated surface area, adhere to the urea surface, exhibiting a gradual release. Furthermore, nano ZnO coatings comprise finely dispersed particles, while bulk ZnO tends to form particle agglomerations, adding another layer of complexity to their respective performance.

In the current investigation, the utilization of nano–sized zinc significantly outperformed its bulk ZnO counterpart in enhancing zinc assimilation within rice tissues. The nanoparticles, owing to their expansive surface area and elevated reactivity, acted as catalysts for superior zinc absorption and bioavailability (Liu et al., 2016). Applying urea coated with ZnO to the soil demonstrated an uptick in the activity levels of both urease and phosphatase enzymes, which likely facilitated a greater soil–based zinc availability, ultimately benefitting the plant’s edible parts (Abdullah et al., 2022). Interestingly, due to their high surface area and improved solubility, ZnO NPs resulted in amplified zinc concentrations within plant tissues when compared to bulk ZnO (Prasad et al., 2012). This higher zinc levels in milled rice might be attributed to the xylem–based translocation of ZnO NPs from the roots to the aerial parts and eventually to the grains (Pérez–de–Luque, 2017). Prior studies have also demonstrated the efficacy of zinc–fortified urea in enhancing zinc uptake in other cereal crops like wheat (Dimkpa et al., 2020) and maize (Pooniya et al., 2017). In this study, a noteworthy anomaly surfaced: lower zinc concentrations were observed in both milled rice and straw from lone urea–treated rice as opposed to those treated with soil–applied bulk ZnO at 5 kg Zn ha^–1^. This phenomenon can likely be rationalized by the dilution effect (Jarrell and Beverly, 1981). Significantly, the straw component served as the principal zinc repository within the rice plant, displaying zinc levels that were multiple times greater than those in grains. Our initial hypothesis, positing an enhanced zinc uptake from zinc–coated urea samples, was corroborated (Liu et al., 2016). Elevated grain yields were attributed to the greater zinc bioavailability from nanoparticle–treated plots compared to controls and bulk ZnO applications. In alignment with our hypothesis, urea coated with 0.2% NZnCU appeared to stimulate the functionality of various plant–based zinc transporters, thereby resulting in augmented zinc levels in both milled rice and straw. Overall, the observed differences across treatments can be credited to both the residual impact of green manuring (GM) on the rice crop, as well as the beneficial influence of zinc fertilization. Thus, the strategic incorporation of ZnO NPs in this study underscores their potential to minimize fertilizer inputs without compromising either crop yield or nutritional integrity.

## 5 Conclusion

Relative to alternative green manuring sources, the physiological growth and development of *Basmati* rice exhibited superior performance when augmented with *Sesbania* as a pre–transplantation green manure. Further enhancement in both grain and straw yields was achieved when this organic manuring strategy was synergistically combined with nano–zinc (NZnCU) fertilization at a concentration of 0.2%. These results suggest that coupling nano–zinc fertilization with the incorporation of summer green manures like Sesbania in *Basmati* rice could enhance growth trajectories and productivity and may offer a robust framework for nutrient management. Such a strategy could be advantageous in intensively–cultivated rice ecosystems, promising not only superior growth and nutritional outcomes but also enhanced economic viability.

## Data availability statement

The original contributions presented in the study are included in the article/**Supplementary Material**. Further inquiries can be directed to the corresponding author.

## Author contributions

KB, YSS, and RP led the research work, planned, supervised, and conducted field experiments, and read and edited the manuscript. KB, SM, SN, and KR collected soil, plant samples and performed chemical analysis, also wrote the initial draft of the manuscript, and prepared figures and tables. RP, YSS, DK and CS project supervision, reviewed, read, and edited the manuscript with significant contribution. All authors contributed to the article and approved the submitted version.

## Supplementary material

Supplementary Figure 1 Dry matter accumulation (t ha^–1^) by summer green manuring crops.
