# Supplementary material for: Enhancing physiological metrics, yield, zinc bioavailability, and economic viability of Basmati rice through nano zinc fertilization and summer green manuring in semi–arid South Asian ecosystem
Source: Front Plant Sci. 2023 Oct 31;14:1283588. doi: 10.3389/fpls.2023.1283588 (PMC10644410; doi:10.3389/fpls.2023.1283588)
Supplement: Supplementary Figure 1 — Dry matter accumulation (t ha–1) by summer green manuring crops. [file DataSheet_1.docx]

Supplementary Figure 1 (S1)

Bar indicates a standard error (±)

FIGURE S1. Dry matter accumulation (t ha^–1^) by summer green manuring crops
